# Evaluating Monaco 6.2.2 in complex radiotherapy across matched LINACs: improved MLC modelling and dose accuracy with virtual source model 2.0

**DOI:** 10.1007/s13246-025-01602-5

**Published:** 2025-07-21

**Authors:** Luis Muñoz, Peter McLoone, Peter Metcalfe, Anatoly B. Rosenfeld, Giordano Biasi

**Affiliations:** 1 Genesiscare Flinders Private Hospital, Bedford Park, SA Australia; 2 South Western Sydney Local Health District, Liverpool, NSW Australia; 3 Centre for Medical Radiation Physics, University of Wollongong, Wollongong, NSW Australia

**Keywords:** MLC, Modelling, Characterisation, SRT, Spine, SIMT

## Abstract

This study assesses the updated Monaco TPS virtual source model (VSM) 2.0, which removes multileaf collimator (MLC) and jaw characterization as editable factors from the MLC geometry section within Monaco. The focus is on the impact of changes to stereotactic radiotherapy (SRT) cases for spinal and intracranial treatments for two beam matched linear accelerators. A validated custom VSM 1.6 model optimized for SRT was compared with the Elekta Accelerated Go Live 6 MV flattening filter-free (FFF) and VSM 2.0. Evaluations included measured MLC characteristics with a high-resolution detector, measured output factors (OPF), ion chamber fields in the thorax phantom, and recalculations of clinically relevant SRT cases. VSM 2.0 improves MLC modelling. Ion chamber measurements for IAEA TD1583 measurements were found to be within expected tolerances. Gamma pass rates for two matched LINACs evidenced improvement at 1%, 1 mm and 10% threshold for single and multi-SRS brain and SABR Spine treatments. VSM 2.0 represents a meaningful advancement in beam modelling within a Monte Carlo-based TPS environment, offering improved dosimetric performance and operational simplicity. Commercially available detectors were used to demonstrate that VSM 2.0 enhances agility MLC modelling, supporting more precise SRT and SABR delivery for matched LINACs. Removing configurable dependencies from the beam model will result in more consistent high quality beam models, an improves workflows for commissioning of the Monaco TPS.

## Introduction

Monte Carlo-based TPS algorithms have enhanced the ability to simulate complex radiation interactions with high fidelity [1]. Accurate dose calculation is fundamental in radiotherapy, achieving this accuracy depends on minimizing the differences between the physical components of medical linear accelerators (LINACs) and their representation in treatment planning systems (TPS). The accuracy of a TPS LINAC model is primarily influenced by the quality of beam data acquisition [2], the precision of MLC and jaw characterization, algorithmic assumptions, machine-specific behaviours, and the fidelity with which these are represented within the dose calculation engine. As highlighted by Smilowitz et al. [3], inaccuracies in the commissioning or characterization of these parameters can propagate through to the final calculated dose, particularly in complex treatment modalities such as stereotactic radio surgery (SRS) with volumetric modulated arc therapy (VMAT).

A critical aspect of beam modelling is the characterization of the beam limiting device (BLD), which includes multileaf collimators (MLC) and jaws [4–7]. In the Monaco TPS (Elekta Oncology Systems, Crawley, UK), the Agility MLC and jaw characterization can be modified through geometry factors within its Virtual Source Model (VSM) 1.6, these are defined within the transmission probability filter (TPF). Accurately replicating the dosimetric behaviour of the BLD remains challenging. Parameters such as leaf transmission, interleaf leakage, leaf offset, and leaf tip leakage often require iterative manual adjustment [8], particularly for clinics treating complex intra- and extracranial SRS cases. Discrepancies may arise because TPS beam models may not fully capture the dosimetric behaviour of MLC due to limitations in detector resolution, commissioning tools, or user experience. Recent studies by Thewes et al. [9] and Hernández et al. [10] emphasize the need for robust characterization of the BLD within VSM 1.6, highlighting limitations in how it accounts for tongue-and-groove. Saez et al. [11] further highlights the importance of standardizing MLC parameter configuration in the TPS to reduce variability between calculated and measured dose distributions to improve consistency across matched machines.

In response, vendors are evolving their dose calculation algorithms to better model MLC behaviour intrinsically, as seen with Elekta’s VSM 2.0 and Varian’s enhanced MLC modelling introduced in Eclipse V16 [12]. Pagani et al. [13] report improved agreement between well-matched LINACs and the TPS, as reported in recent validation studies in the Eclipse space. The Agility MLC presents a unique challenge owing to its defocused leaf bank design, which aims to reduce tongue and groove effects. Trade-offs are required to model certain physical MLC aspects within the TPF, and reliance on geometric factors can introduce potential inaccuracies. The process of adjusting these geometry values is iterative and can be time consuming. Hernandez [10] underscores the importance of optimizing MLC parameters based on specific treatment plan characteristics and highlighted the limitations of VSM 1.6 and RayStation in accurately reproducing the MLC characteristics of the Agility. Monaco version 6.2.2 introduces VSM 2.0 with graphics processing unit (GPU)-accelerated calculation, with the removal of the conventional user defined geometry factors for MLC and jaw characterization. This updated model aims to reduce user dependence and ad hoc corrections to the MLC and jaw characterization.

The purpose of this study is to evaluate the performance of VSM 2.0 within the context of complex stereotactic treatment plans including spine, single-target and single isocentre multi-target (SIMT) brain stereotactic radiosurgery (SRS). The Elekta Accelerated Go Live (AGL) 6 MV Flattening Filter Free (FFF) beam model will be compared against our institutions custom 6FFF VSM 1.6 model with optimised geometry factors. Notably, two beam-matched Elekta LINACs were used to control for machine-specific differences, isolating the impact of the beam model in the comparison. Through gamma pass rate (GPR) and point dose analyses, this study investigates whether VSM 2.0 delivers consistent and robust dosimetric performance across matched LINACs in the context of high-complexity SABR and SRS plans when compared to a validated 6FFF model in VSM 1.6 specifically characterized for complex treatments. To complete this, we utilized a commercially available high-resolution detector, routine commissioning equipment and standard clinically relevant complex plans.

## Methods

### Institutional setup

Our institution uses a matched LINAC model of Elekta VersaHD LINACs with Agility MLC for conventionally fractionated and hypofractionated treatments. These LINACs are matched according to the Rijken criteria [14], with agreement in scanned profiles to within 1.0%, percentage depth-dose (PDD) points within 0.5% and field output factor (OPF) within 1.0% of measured down to a 1.0 × 1.0 cm^2^ field, where tolerance is set at 2.0% [15, 16]. A subset of these LINACs, specifically equipped with a HexaPOD 6 degrees of freedom couch and BrainLab (BrainLAB AG, Feldkirchen, Germany) ExacTrac or C-RAD (C-Rad, Uppsala, Sweden) Catalyst HD + surface guidance, are dedicated to stereotactic radiotherapy (SRT) procedures for non-coplanar couch delivery. LINAC performance is maintained with mechanical and dosimetry tolerances set by AAPM TG 142 [17], and MPPG 8.b [18] which provides minimum practice standards for QA programs. Radiation isocentre is monitored using routine Winston-Lutz for kV to MV isocentre to ensure these coincide to within 0.25 mm over the full gantry rotation range [19]. Isocentric couch rotation is maintained within1.0 mm from 270 to 90 degrees, in accordance with recommendations outlined in MPPG 8.b [18] and SRS-SBRT-specific guidance from AAPM MPPG 9.b [20]. These guidelines emphasize the importance of precise small-field dosimetry, comprehensive end-to-end validation, and stringent quality assurance practices to achieve a targeting accuracy of ≤ 1.0 mm.

### Improvements to Monaco TPS: VSM 1.6 versus VSM 2.0

#### Existing clinical model: VSM 1.6

The clinical 6 MV FFF model in Monaco 6.1.4 utilizes VSM 1.6, and the MLC geometry values in the TPF to characterize the BLD are detailed in Table 1, where most of the factors are left at their default values. This model has been specifically optimized for SRT, including intra- and extra-cranial body sites [21, 22].

**Table 1:** Monaco 6.1.4—virtual source model 1.6 6FFF geometry TPF values

Default value	Parameter	Value
✓	Static leaf gap (mm)	0.1
✓	Leaf transmission	0.003
	Leaf groove width (mm)	0.5
	TJaw transmission	0.006
✓	PJaw transmission	1.0
	Interleaf leakage	7.0
	Leaf tip leakage	1.15
✓	TJaw tip leakage	1.03
✓	PJaw tip leakage	1.0
✓	TJaw plane position (mm)	432
✓	PJaw plane position (mm)	317.7
✓	TJaw backscatter	0
✓	PJaw backscatter	0
	Leaf offset (mm)	− 0.05

#### Agility accelerated go live model: VSM 2.0

The model used to validate the introduced modifications was the Elekta Accelerated Go Live (AGL) 6FFF model provided by the vendor. This model is derived from both scanned and non-scanned data acquired by Elekta. Monaco 6.2.2 incorporates a GPU-based Monte Carlo algorithm for photon energies of 6 and 10 MV, with and without a flattening filter. VSM 2.0 introduces a significant change by eliminating the geometry factors previously used to characterize the MLC and jaws. As a result, post-modelling corrections are no longer necessary for clinics using the vendor-supplied Agility configuration. Collectively, this update aims to reduce discrepancies between calculated and measured doses, ultimately improving treatment accuracy.

#### Updated clinical workflow in the absence of geometry-based modelling parameters

**Fig. 1:**
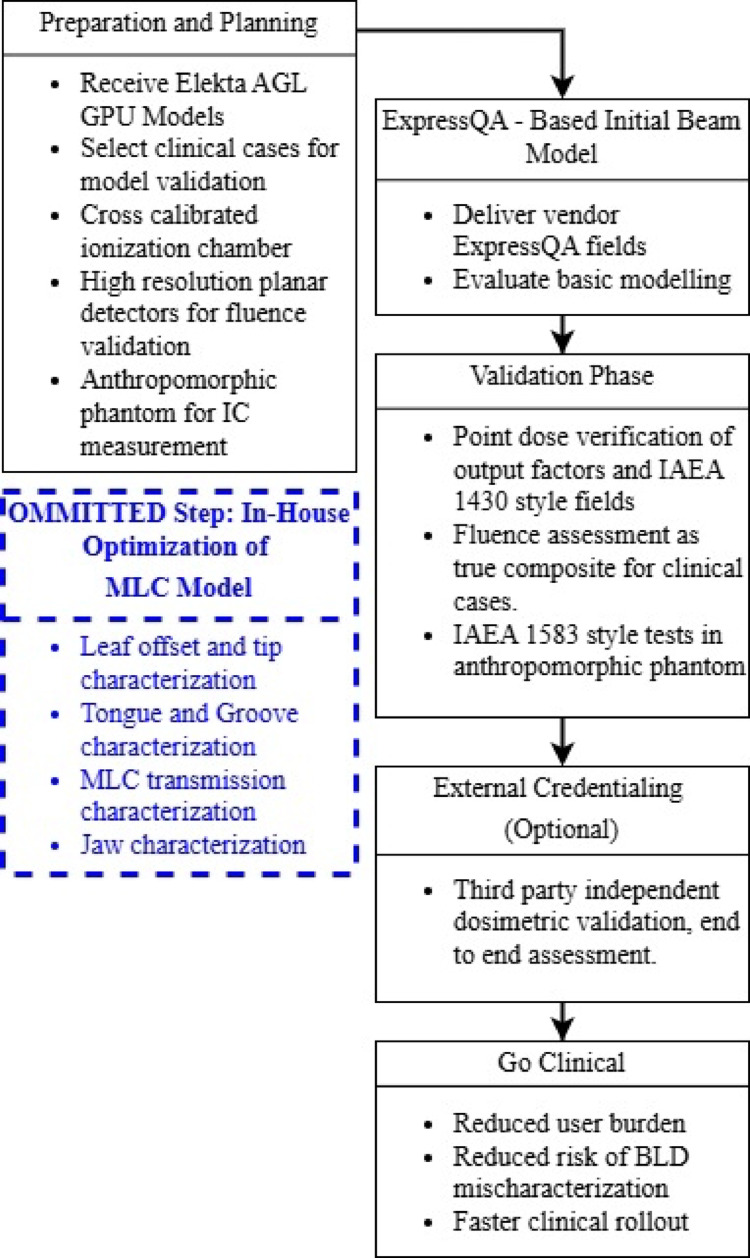
Clinical validation workflow for VSM 2.0 model without geometry factors

The introduction of VSM 2.0 allows for a streamlined workflow towards clinical model release. Historically, commissioning workflows such as those described by Snyder et al. [7] and Roche et al. [6] included extensive physical characterization of the geometry parameters using planar detectors, film dosimetry, and point-dose measurements to iteratively optimize the TPF and MLC model. These methods required balancing parameter sets that could overfit QA field profiles while potentially degrading clinical plan accuracy. In contrast, VSM 2.0 reduces the risk of over-modelling and removes the need for tuning of geometric fluence components. Consequently, Fig. 1 details a clinical workflow shift to validate the vendor-supplied model through a limited set of the ExpressQA fields, point-dose measurements, using OPF and anthropomorphic phantoms and representative clinical test cases. This streamlined process not only decreases user burden but also enables faster clinical rollout while preserving dosimetric accuracy for clinically relevant geometries.

### Beam limiting device validation using ExpressQA fields

**Fig. 2:**
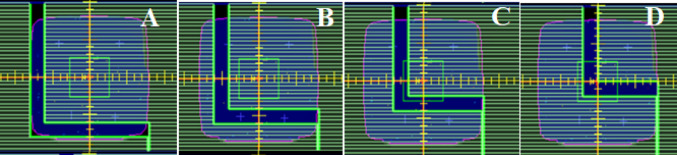
Four-L sequence, where progressive deliveries expose an L shape nested in the previous delivery running from shape **A** to **D**

**Fig. 3:**
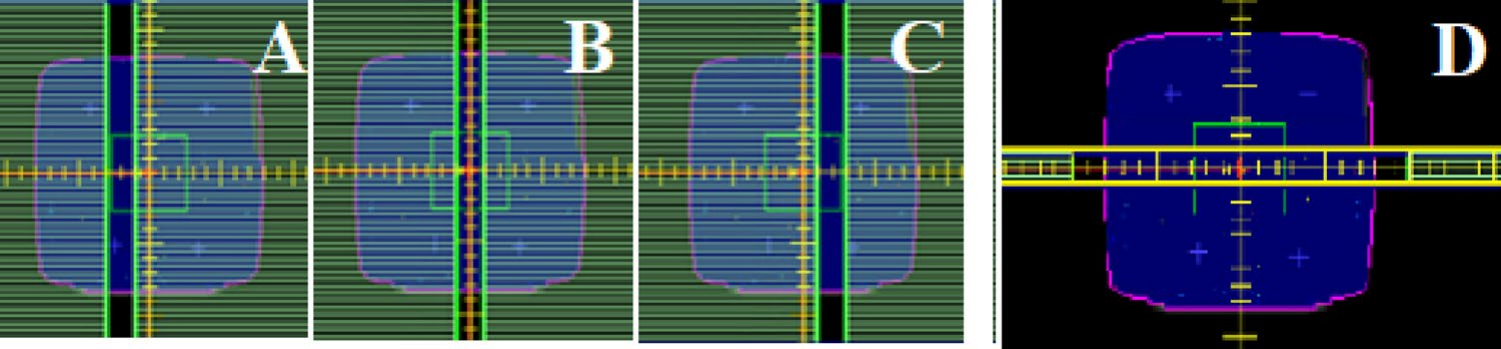
3 Segment abutting sequence, where progressive deliveries expose a 2.0 cm strip across the detector from shape **A** to **C**. Figure **D** is the jaw only characterization field that collimates with Y1 and Y2 jaw only

Historically, the Express QA fields provided by the vendor have been used to optimize the MLC geometry factors in the TPS. Key MLC characteristics influencing plan quality include leaf offset, leaf tip leakage, and transmission [6, 7, 9]. Three fields would be used to evidence characterization improvements in VSM 2.0 for MLC and jaws are: the FourL (Fig. 2) and 7SegA (Fig. 3A–C) detail leaf groove characteristics (tongue and groove), transmission properties and MLC minor and major offset. The Y-Jaw only (Fig. 3D) field validates jaw characterization, ensuring accurate modelling of jaw positioning and transmission. These fields provide a comprehensive assessment of the MLC and jaw characteristics and can be leveraged as a preliminary tool to test modelling accuracy (Post Modelling Adjustment of MLC Parameters, Version 4.00, Document ID: LRMMON0003). VSM 2.0 assessment was completed by delivering plans to the Sun Nuclear SRS MapCheck (Sun Nuclear Corporation) which has a detector spacing of 2.47 mm within an array of 77 mm × 77 mm for a high-resolution assessment, calculations used a grid of 1 mm and statistical uncertainty of 0.5%, per control point. While the SRS MapCHECK has a smaller active area compared to film, it offers a reliable and practical alternative for evaluating high-gradient dose regions. As demonstrated by Stedem et al. [23], the device provides comparable sensitivity to film in detecting clinically relevant dose discrepancies, especially when using tight gamma criteria. Importantly, the array enables consistent characterization of multileaf collimator (MLC) and jaw behaviour without the handling and processing uncertainties associated with film dosimetry.

### Benchmarking simple to complex cases: VSM 2.0 versus VSM 1.6

A comparative validation of VSM 2.0 versus VSM 1.6 performance was conducted following guidance from AAPM MPPG 5.b [24], combining both simple and complex test cases. For baseline system validation, output factors and open field tests were performed using a thorax phantom in accordance with IAEA Technical Document 1583 [25], with measurements acquired using a calibrated ionization chamber. This approach provided a simplified yet robust verification of LINAC-TPS agreement. To evaluate complex modulated delivery, VMAT SRS and SABR plans were benchmarked using representative clinical cases designed in the style of AAPM TG-119 [26]. These plans were delivered across a network of SRS/SABR-capable machines and validated against 2D fluence measurements using a high-resolution detector in the SRS MapCHECK. This combined methodology enabled assessment of both fundamental dosimetric consistency and the system’s performance under clinically relevant, high-complexity conditions, thereby addressing both the basic and advanced requirements of TPS and LINAC validation.

#### Non-scanned validation—OPF measured vs calculated

The measured OPF were acquired on LINAC 1 within a Blue Phantom II water tank using a PTW microdiamond detector, the detector was set at a depth of 100 mm with a surface to source distance of 900 mm. Recommended detector correction factors were applied for small fields < 3.0 cm after centring the detector on the central axis for measurement [27]. Fields were collimated with MLC in cross plane and jaws in the in-plane from 10 × 10 mm to 400 × 400 mm and were acquired to compare with the VSM 2.0 and VSM 1.6 calculated results. Calculation of OPF used for comparison against measured were completed using a 1 mm grid and 0.5% statistical uncertainty per calculation in a solid water phantom. OPF were gathered for each field using the mean dose to a sphere of radius 0.17 cm which roughly represents the volume of the CC01 ionization chamber [28]. Additionally, these values were averaged from three separate imports of a water phantom patient into the TPS to generate a new Monte Carlo seed point for each calculation [22].

#### Calculation of point doses in thorax phantoms—TecDoc 1583

IAEA TecDoc 1583 methodologies within a CIRS thorax phantom (CIRS Inc., Norfolk, VA, USA) were implemented to verify the dosimetric consistency between measured data from LINAC 1, VMS 2.0 and VSM 1.6. These evaluations validate the TPS performance utilizing measured data acquired using an IBA (Schwarzenbruck, Germany) model IC13 chamber (SN11596). The incorporation of ionization chamber measurements in the assessment was deemed appropriate, as they are considered the gold standard measurement instrument, providing reproducible point dose measurements within a heterogeneous phantom.

#### Validation of beam model changes—calculating clinically representative cases

SRT provides non-invasive treatment for unresectable tumours in both the brain and body and has become the standard of care for medically inoperable patients [29–31]. Plans can be produced with highly modulated sequences based on body site, proximity, number, and size of target(s), leading to increased complexity compared to conventional radiotherapy. The essential requirements of SRT for safe and effective treatment include adherence to tighter mechanical and dosimetric tolerances assigned to the treatment system [17, 20, 32].

Benchmark plans used for testing LINAC performance across our institution for commissioning would be used to validate the matched-ness of our LINACs to the AGL 6FFF VSM 2.0 model, and our well curated and validated set of geometry factors set within VSM 1.6 [33].

Intracranial plans employed at most three non-coplanar couch kicks for beam delivery and included apex fields at 270°or 90°, each comprising to 3–6 beams (Table 2). The choice of evaluating the average segment size and MU efficiency served as indicators of plan complexity for the brain cases, offering insights into the modulation and efficiency of dose delivery. The spine cases included at most three beams delivered in a co-planar manner (Table 2). Given that characteristics such as average segment size or minimum aperture opening lacked sufficient discriminatory power in detailing complexity, MU and MU efficiency were the preferred delineators of complexity.

**Table 2:** List of single and multi-target cranial benchmarking cases

Plan [#]	PTV [#/tot]	PTV coordinates [mm]	PTV volume [mm^3^]	PTV dose [cGy]	Average segment size [cm^2^]	MU/cGy
1a	1/1	(0,0,0)	20	500	3.62	2.14
1b	1/1	(0,0,0)	10	1000	1.73	2.03
1c	1/1	(0,0,0)	8	2000	1.31	2.02
2a	1/1	(3.0, 0, − 0.04)	20	1000	3.28	2.51
2b	1/1	(3.0, 0, 0)	8	1000	0.61	3.14
3a	1/1	(− 0.04, 6, 0)	20	1000	3.31	2.39
3b	1/1	(− 0.04, 6.1, 0)	8	2000	1.46	0.99
7a	PTV Adj. brainstem	(0,0,0)	15	1800	2.67	2.25

					MU per beam	MU/cGy per beam
5	1/5	(− 4.1, 1.4, − 2.0)	10–30	2000	2691	1.35
5	2/5	(1.8, 0.4, 3.3)			2022	1.01
5	3/5	(4.7, − 1.1, 0.6)			2357	1.18
5	4/5	(− 4.6, − 1.6, 2.8)			2339	1.17
5	5/5	(2.1, 0.7, − 3.3)			2214	1.11
					2290	1.15

Plan 1 (a, b and c) delivered to isocentre; plan 2 (a, b) delivered at 3 cm off axis in the cross-plane direction; plan 3 (a, b) delivered 6 cm off axis in the in-plane direction. Plan 7a is a plan on central axis, target abutting the brainstem. Plan 5 is a 5-target plan of target size ranging 10–30 mm^3^

The plans used clinically relevant cost functions to ensure that the optimization engine would perform as clinically intended. Among the sequencing parameters used to influence the TPS optimizer, the minimum segment width, VMAT beam increment and fluence smoothing are regarded as important factors in the post-fluence optimization. These were set to 0.6 cm, an increment of 20, and high to help restrict the amount of modulation and plan complexity. A maximum of 100 control points per arc were also used to help constrain the overall complexity of the segmentation performed by the optimizer. These plans are by no means exhaustive; however, they aim to capture an indicative set of clinically relevant cases (Table 3).

**Table 3:** List of clinically representative spine plans for specific sites

Plan [#]	Vertebra	# Beams	PTV dose [cGy]	MU	MU/cGy
1	T7	2	900	4654	5.17
2	T8	1	900	4643	5.16
3	L1	2	600	3135	5.23
4A	L1	1	900	5333	5.93
4B	T10	1	900	5289	5.88
5	L4	1	800	3943	4.93
6	T8	1	900	5440	6.04
7A	T5	1	800	4683	5.85
7B	L3	1	900	3565	3.96
8	L4	1	900	6000	6.67

### Measuring test cases

**Fig. 4:**
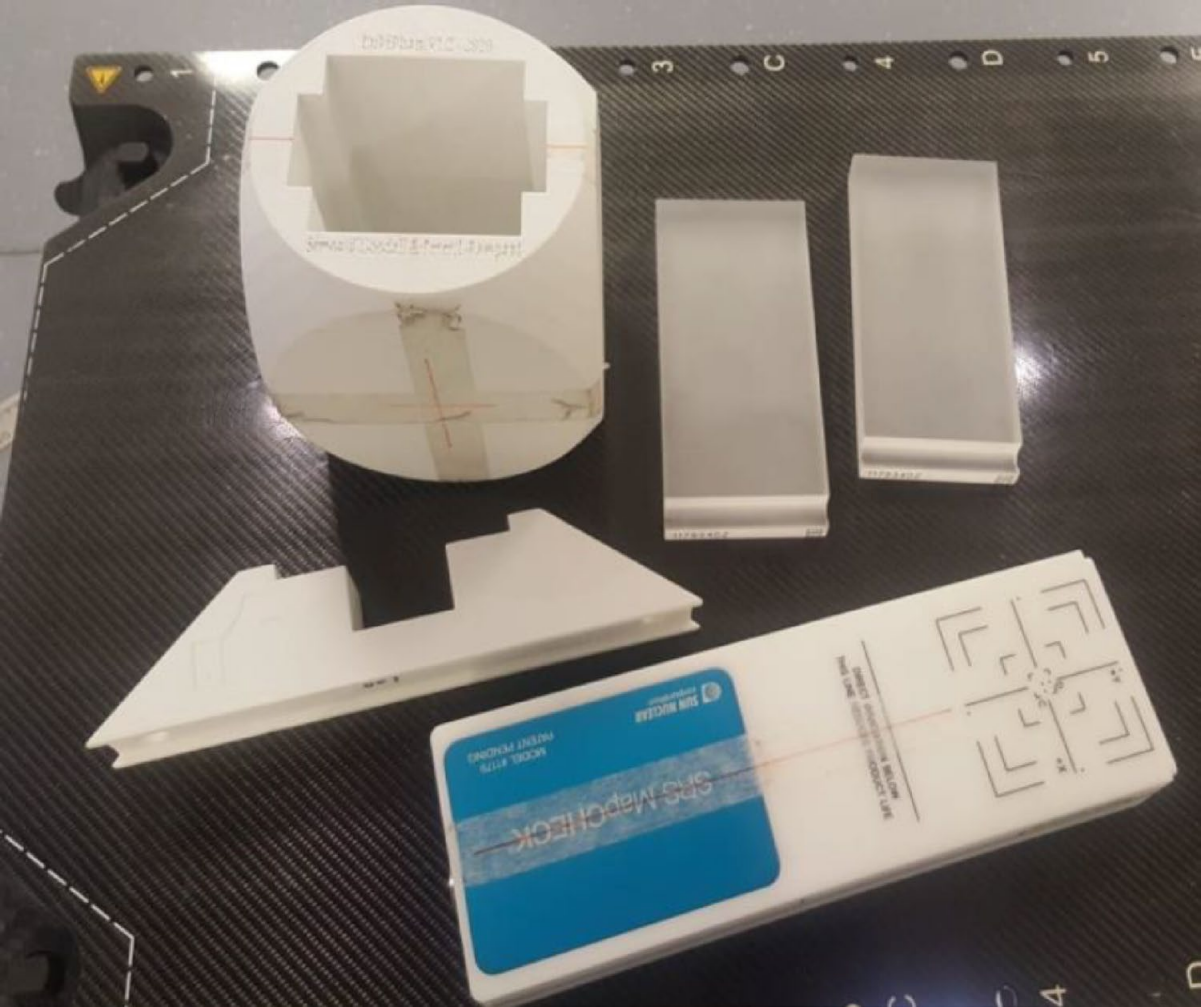
SRS MapCHECK and custom 3D printed holder and Perspex inserts

The measurements were carried out on two nominally matched Versa HD LINACs using an SRS MapCHECK device (Sun Nuclear Corp, Melbourne, FL, USA) within a custom tissue-equivalent cuboid phantom (Fig. 4). The SRS MapCHECK has demonstrated near-equivalence with films for patient-specific QA in SRS and SBRT cases for real time measurement [34]. Recent work by Stedem et al. [23] has further validated the device’s clinical utility, demonstrating that its performance satisfies the Nyquist sampling requirement for the spatial frequencies typically encountered in stereotactic fields. This confirms the arrays adequacy in capturing high-gradient dose distributions critical for accurate gamma analysis in SRS QA. The phantom was positioned at the isocentre using a reference 5.0 × 5.0 cm field, which was delivered from gantry zero and HexaPOD. This included the use of the HexaPOD reference frame to verify the non-coplanar couch angles for intracranial SRT. For spine cases, co-planar beams are delivered with the SRS MapCHECK oriented in the sagittal plane to sample the transition across the target and spinal cord.

The comparison between planned and measured dose distributions for both LINACs was carried out using gamma analysis with a range of criteria: 3%, 1 mm; 2%, 1 mm; and 1%, 1 mm, with a 10% dose threshold. This gamma evaluation was performed using SNC Patient V8.4.1 build 2 software.

To assess the accuracy of the VSM 2.0 vs VSM 1.6 across two LINACs, we performed a statistical analysis of the gamma pass rates at 1% and 1 mm (10% threshold) for each model under three criteria. For the SRS brain cases, a Two-Way ANOVA with replication was employed to examine the effects of segment size and model type on the gamma pass rates and to determine whether an interaction existed between these factors. Segment sizes ranging from 0.61 to 3.62 cm^2^ were used, with gamma pass rates calculated for both models across all criteria (3%, 1 mm; 2%, 1 mm; and 1%, 1 mm).

For the SIMT case, the effect of the distance from the isocentre was considered with respect to the model and gamma pass rate. For SABR spine cases, the modulation factor, defined as monitor units per centi-gray (MU/cGy) was reported, which is used as an indication of the complexity of the beam modulation for each treatment plan. This factor allows for a more detailed comparison of the gamma pass rates between cases with factors ranging from 3.96 MU/cGy to 6.67 MU/cGy.

## Results

### Output factors

**Fig. 5:**
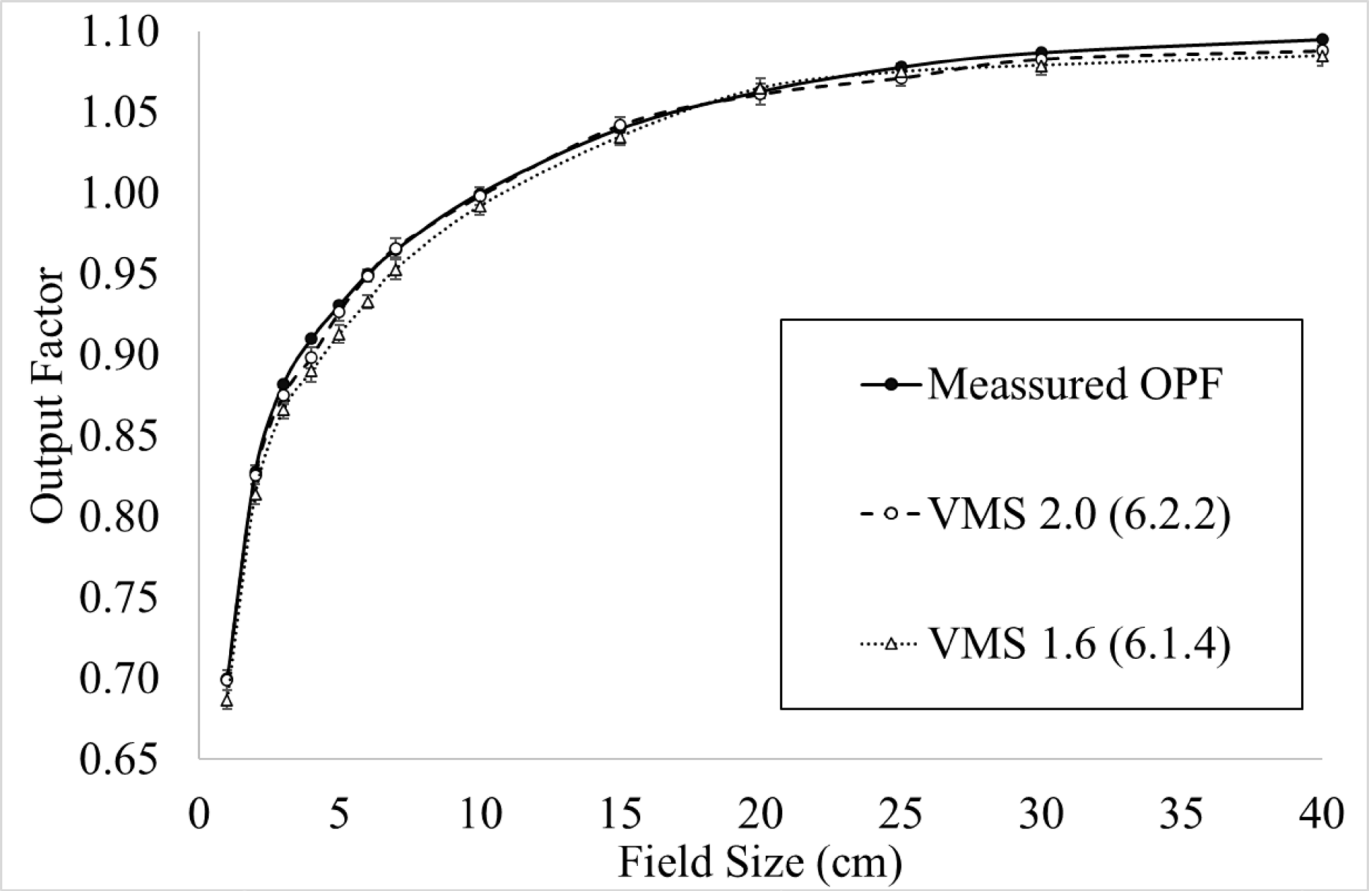
Output factors for measured and calculated effective field size. Measured OPF were completed with Measured, VSM 2.0 and VSM 1.6

Figure 5 presents the measured and calculated OPF as a function of field size for VSM 1.6 (Monaco 6.1.4) with optimized leaf tip leakage and leaf offset geometry factors (Table 1), and VSM 2.0 (Monaco 6.2.2). The measured OPFs (solid line) are compared against TPS-calculated OPFs for both beam models, where error bars indicate the standard deviation of the calculated values. Across all field sizes, VSM 2.0 (open circles, dashed line) demonstrates improved agreement with measured data compared to VSM 1.6 (open triangles, dotted line), particularly for small fields. Both models converge for larger field sizes, where differences are within 1% for a 400 × 400 mm field. The calculated OPF exhibit low variability, as reflected by the small error bars.

### MLC characteristics

Gamma analysis was employed to quantitatively assess the dosimetric improvements between the VSM 2.0 and VSM 1.6 MLC shapes (Table 4). The FOURL and 7SegA fields were particularly effective in resolving discrepancies related to leaf groove structure, offset calibration, and transmission adjustments, whereas the Y-Jaw Only field validated jaw characterization.

**Table 4:** Pass rates for MLC characterization fields under VSM 1.6 and VSM 2.0 using a 3%/1 mm and 1%/1 mm gamma analysis criterion

ExpressQA field	VSM 1.6 @ 3%/1 mm	VSM 1.6 @ 1%/1 mm	VSM 2.0 @ 3%/1 mm	VSM 2.0 @ 1%/1 mm
7SegA	84.9	61.1	91.9	71.4
FourL	86.7	56.0	95.0	81.5
YJaw only	100.0	97.4	100.0	98.4

**Fig. 6:**
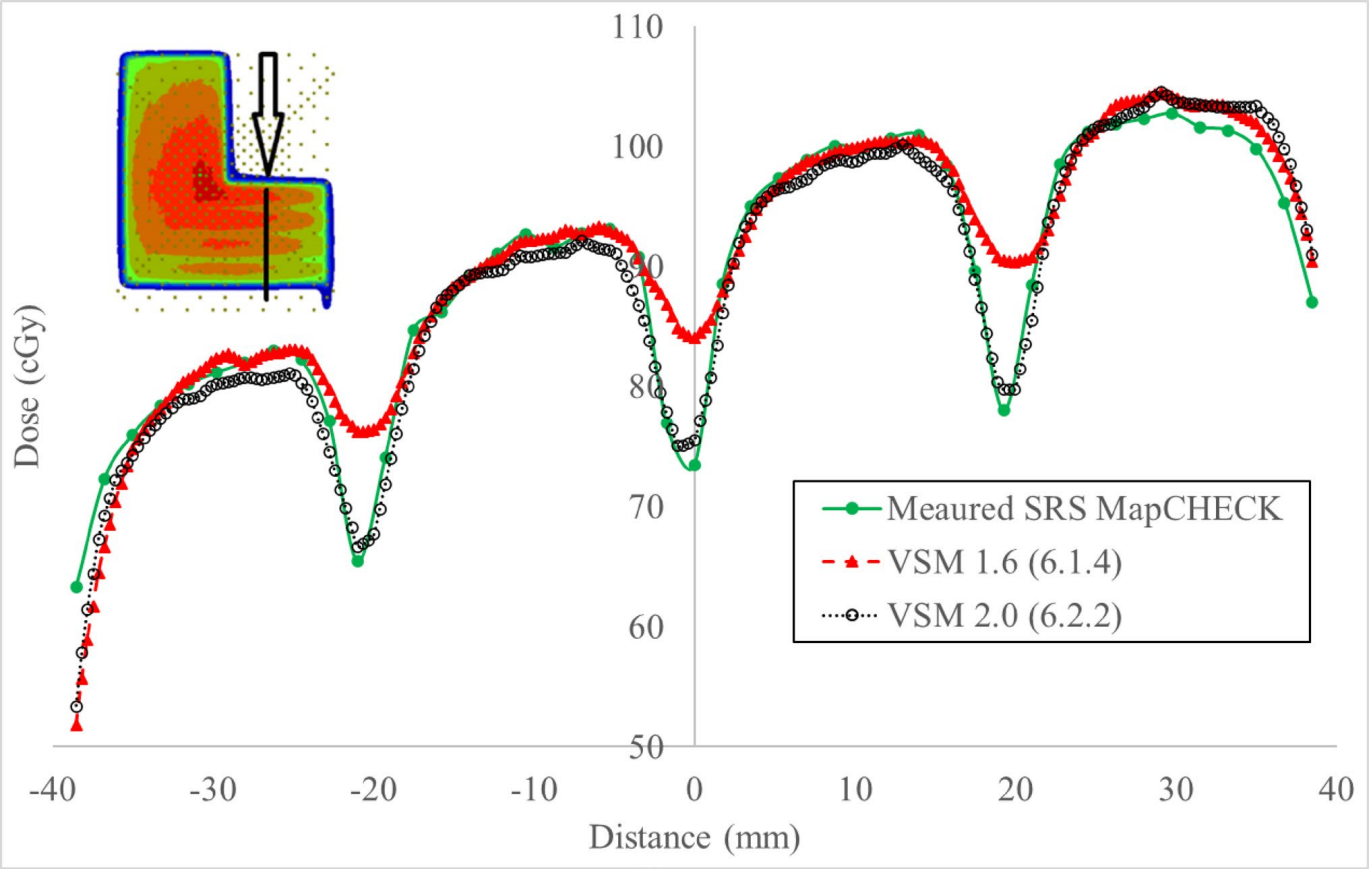
Dose profile across the in-plane direction through the leaf groove region as detailed by the black line on the inset image for measured versus VSM 1.6 and VSM 2.0

Figure 6 details the profiles perpendicular to the travel of the MLC, characterizing the beam model between leaves. VSM 2.0 details improved modelling between leaves. Table 2 details an improvement from 86.7% to 95.0%.

**Fig. 7:**
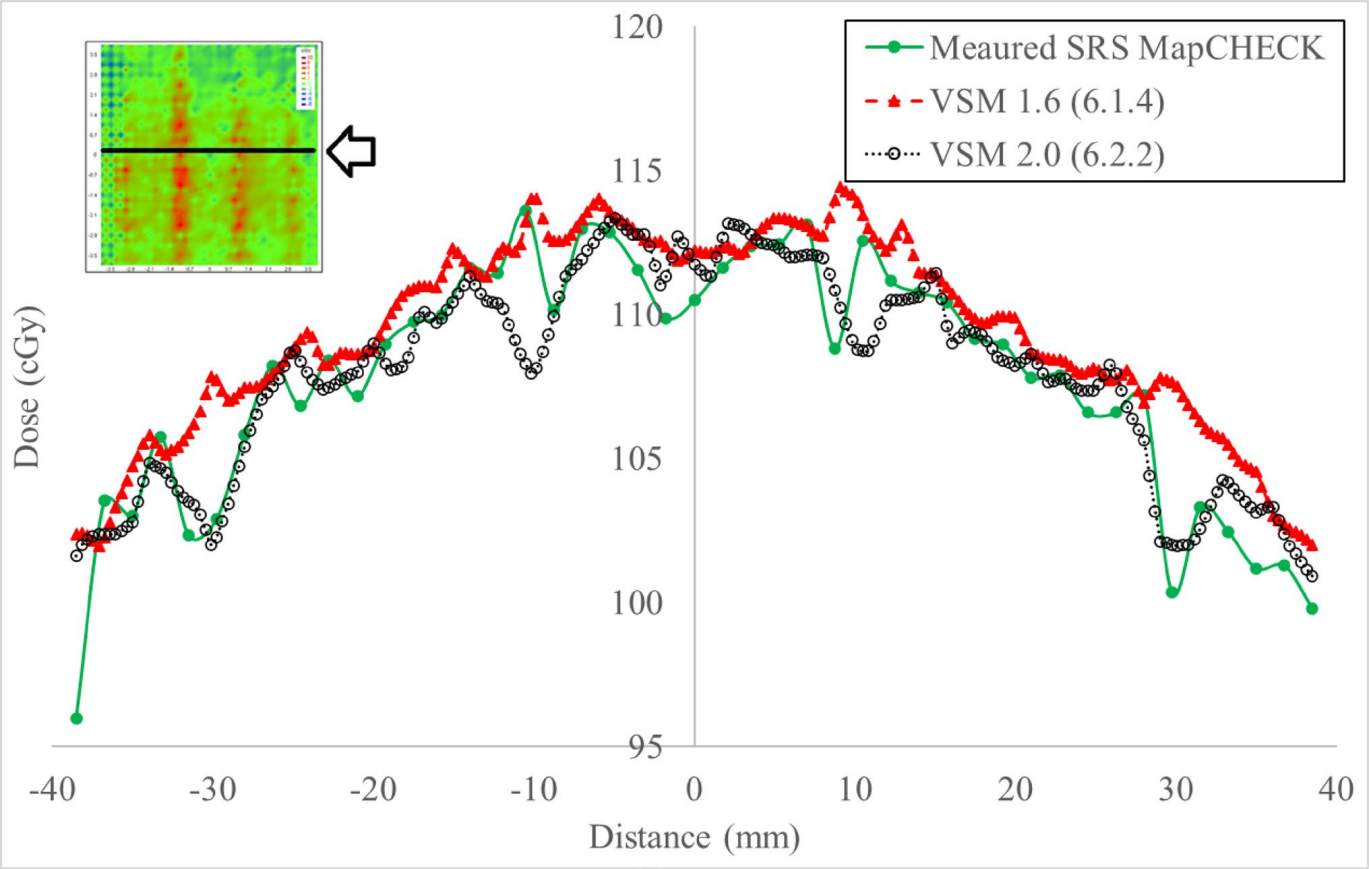
7SegA picket fence field measured versus VSM 1.6 and VSM 2.0 calculated

Figure 7 details 7SegA which is a picket fence style that validates the major and minor off set of the MLC, this field is influenced by the leaf offset and leaf tip leakage in VSM 1.6. Our institution does not apply any physical leaf offsets to an Agility MLC calibration on our LINACs. Improvement in the agreement between measured and calculated is observed in Table 4 for the 7SegA field.

**Fig. 8:**
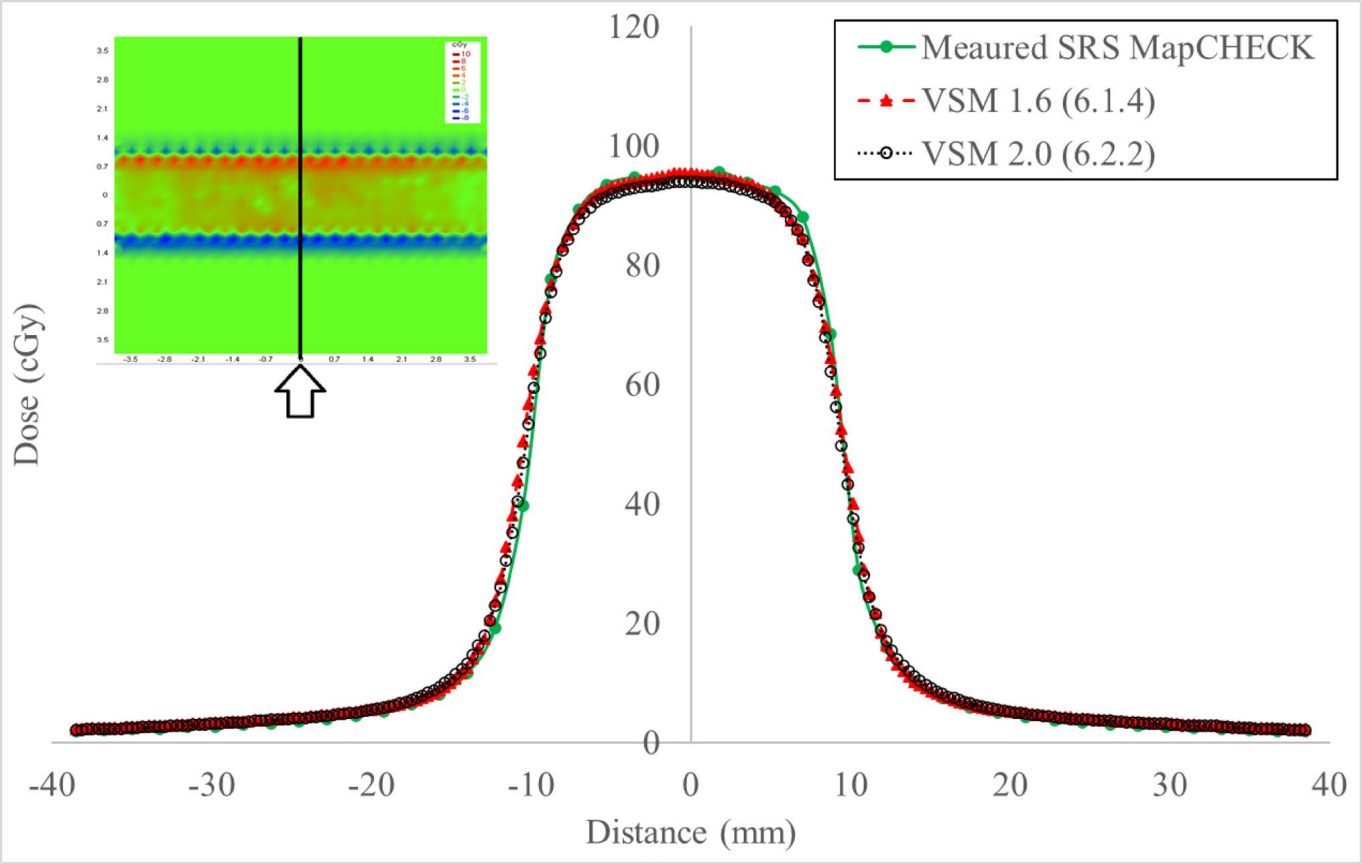
Y Jaw only field for comparison between measured and calculated for VSM 1.6 and VSM 2.0

Figure 8 details a profile across the measured profiles determined by the Y-Jaw only field showing 100% agreement at 3%, 1.0 mm and 10% threshold against calculated.

### Beam model validation with TecDoc 1583

The comparison between LINAC 1, VSM 2.0 and VSM 1.6 showed generally good agreement across test cases, with differences in dose measurements at various reference points typically within ± 2% for most data points. Percentage differences represent the deviation of each model’s calculated dose from the measured dose. Tolerance thresholds indicate the acceptable range for deviations as per IAEA TD1583. Cases 2 and 5 are not measured within our institution as wedges are not used clinically.

**Fig. 9:**
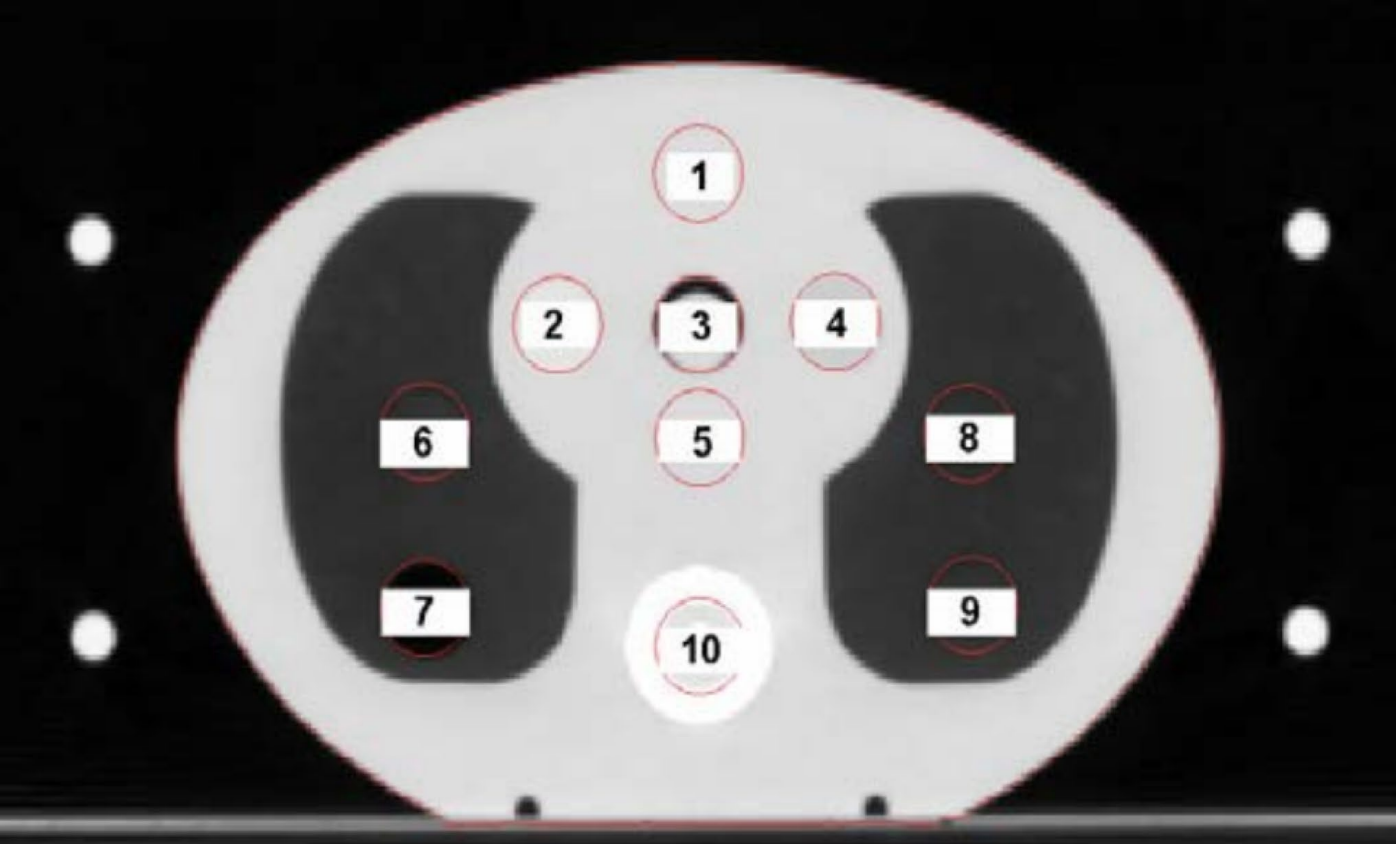
CIRS thorax phantom and measurement positions in tissue, lung and bonelike structures

Figure 9 details the CIRS thorax phantom for measurement position, in Case 1, the percentage difference between measured doses and the two versions varies from -1.4% to 1.0%, with both versions performing within the 2–4% tolerance range (Table 5). Case 3 demonstrates minor deviations, with the largest percentage difference being 2.0% for VSM 2.0 and 1.4% for VSM 1.6 (Table 6). Case 4 shows the smallest deviations in the percentage difference, with differences consistently below 1.4% within lung-like tissue (Table 7). Case 7 highlights a 2.0% difference for both versions, yet still within the acceptable tolerance limits (Table 9). At most a 2.0% difference from measured is observed across the measured doses comparing LINAC 1 with VSM 2.0 and 1.6.

**Table 5:** Measured dose values at selected reference points compared to calculated dose differences using two versions of VSM 1.6 (Monaco 6.1.4) and VSM 2.0 (Monaco 6.2.2)

	Ref point	Measured dose	VSM 1.6 (6.1.4)	VSM 2.0 (6.2.2)	Tol. %
		Dose (cGy)	%Diff	%Diff	
Case 1	1	288.8	0.8%	0.9%	2
	3	234.9	0.9%	0.7%	2
	5	201.2	0.1%	− 0.8%	2
	9	12.3	1.0%	0.7%	4
	10	147.7	− 0.3%	− 1.4%	3

**Table 6:** Case 3 measured dose values at selected reference points compared to calculated dose differences using two versions of VSM 1.6 (Monaco 6.1.4) and VSM 2.0 (Monaco 6.2.2)

	Ref point	Measured dose	VSM 1.6 (6.1.4)	VSM 2.0 (6.2.2)	Tol. %
		Dose (cGy)	%Diff	%Diff	
Case 3	5	51.0	0.1%	− 0.7%	2
	5	45.9	1.2%	2.0%	3
	5	50.2	1.0%	− 0.8%	3
	5	49.8	1.2%	0.2%	3
	6	2.7	0.4%	0.4%	4
	6	3.4	0.0%	0.2%	4
	6	33.5	− 0.2%	− 1.0%	3
	6	66.2	1.4%	1.3%	3
	10	37.8	− 0.7%	0.1%	3
	10	65.6	0.5%	1.5%	3
	10	3.1	0.7%	− 0.1%	4
	10	3.2	0.4%	0.2%	4

**Table 7:** Case 4 measured dose values at selected reference points compared to calculated dose differences using two versions of VSM 1.6 (Monaco 6.1.4) and VSM 2.0 (Monaco 6.2.2)

	Ref point	Measured dose	VSM 1.6 (6.1.4)	VSM 2.0 (6.2.2)	Tol. %
		Dose (cGy)	%Diff	%Diff	
Case 4	3	203.2	0.2%	0.1%	3
	7	112.4	− 1.4%	− 1.3%	4
	10	12.9	1.1%	0.9%	5

### Beam model validation with SRT cases

To validate VSM 2.0 against VSM 1.6 across two matched LINACs and various stereo treatments, box plots were generated for beam model comparisons for each site. These boxplots display results for 3%, 1 mm, 2%, 1 mm, and 1%, 1 mm for plans delivered to the SRS MapCHECK for both models. The box plots illustrate the variability in gamma pass rates, with the median determined by the grey line in each graph. The top and bottom edges of the box indicate the 75th (Q3) and 25th (Q1) percentiles, respectively, forming the interquartile range. The whiskers extend to the most extreme data points that fall within 1.5 times the IQR above and below the box. Data points outside this range are considered outliers and are plotted individually.

Our institution defines plan acceptability as achieving a gamma pass rate GPR of ≥ 95% using 3% and 1 mm at a 10% dose threshold. All plans assessed with VSM 2.0 and VSM 1.6 were considered a pass optimal at this criterion and were considered clinically acceptable. To evaluate the performance differences between VSM 2.0 and VSM 1.6 on two beam-matched LINACs, a series of analysis of covariance (ANCOVA) tests were conducted. Dependent values considered were segment size for the single target SRS cases, distance from the isocentre for the SIMT case and modulation complexity (MU/cGy) for the spine cases. These dependencies were assessed against gamma criteria on pass rates for two matched LINACs. These plans are clinically representative, however not exhaustive.

**Fig. 10:**
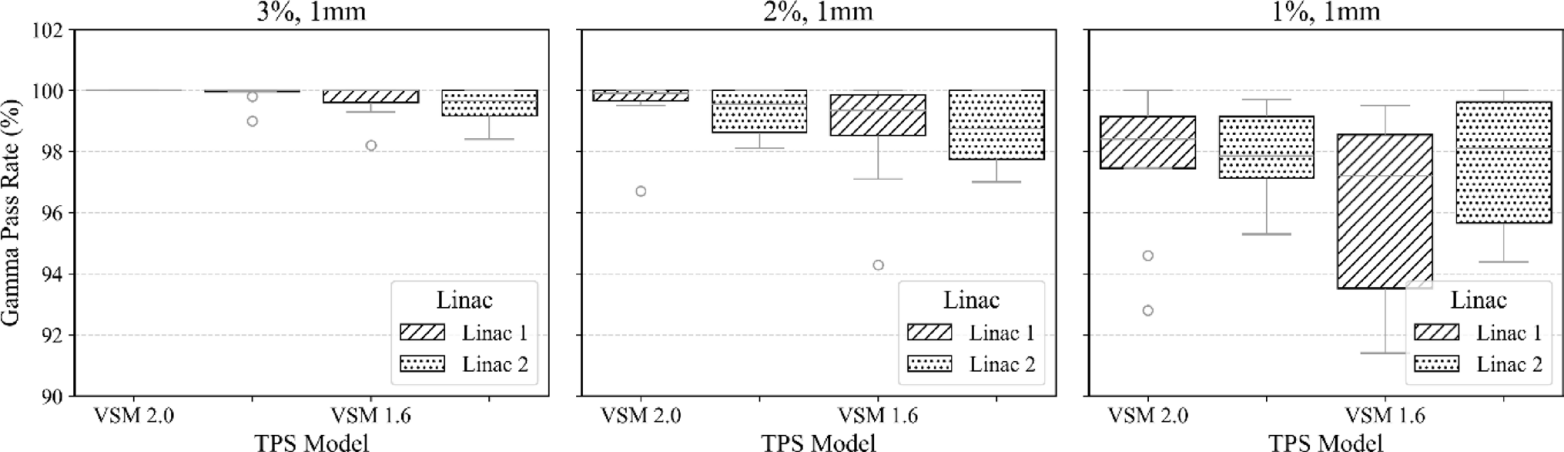
This figure illustrates boxplots that compare gamma pass rates for single-target brain cases, calculated using VSM 2.0 and VSM 1.6, as assessed with the SRS MapCHECK on LINAC 1 and LINAC 2. The results encompass cases delivered on the central axis, 30 mm off-axis in the cross-plane, and 60 mm off-axis in the in-plane

Results from Fig. 10 were analysed to compare VSM 2.0 and VSM 1.6 across two LINACs at the 1%, 1 mm GPR, with average segment size (in cc) as a covariate. The analysis revealed a substantial main effect of the TPS model on GPR, with VSM 2.0 showing higher pass rates than VSM 1.6 (F(1, 28) = 6552.46, *p* < 0.0001). A main effect of LINAC was also observed, with GPR differing between LINAC 1 and LINAC 2 (F(1, 28) = 3078.15, *p* < 0.0001). In contrast, average segment size had no discernible effect on GPR (F (1, 28) ≈ 0.00, *p* = 0.999), and no significant interaction was found between TPS model and segment size (F(1, 28) = 2.07, *p* = 0.15). These results indicate that GPR is primarily influenced by the choice of TPS model and LINAC, rather than plan complexity as measured by segment size.

**Fig. 11:**
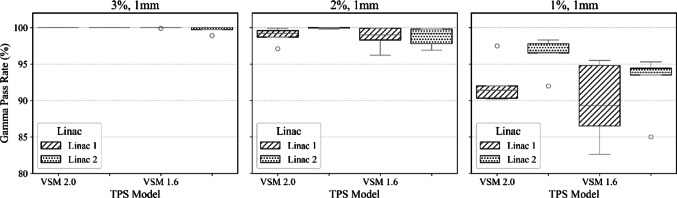
The figure illustrates boxplots depicting the gamma pass rates for measurements conducted at 3%, 1 mm; 2%, 1 mm; and 1%, 1 mm, with a 10% threshold. The results pertain to the five measured targets within a single stereotactic SIMT plan, evaluated for a case calculated using VSM 2.0 and VSM 1.6 for LINAC 1 and LINAC 2

An ANCOVA analysis of Fig. 11 results at the 1%, 1 mm GPR was completed, while controlling for distance from isocenter as a covariate. The interaction between VSM model and LINAC was statistically significant (F(2, 14) = 51.14, *p* < 0.0001), indicating that the performance of the VSM model varied depending on the LINAC used. However, there was no significant main effect of VSM model (*p* = 1.0) or LINAC alone (*p* = 1.0), suggesting that differences were driven by their interaction rather than individual effects. Distance from isocenter did not have a significant effect on gamma pass rates (F ≈ 0, *p* = 0.999), and there was not a significant interaction between the VSM model and distance from isocentre (F = 2.74, *p* = 0.099), though the latter approached marginal significance. These findings suggest that while distance from isocenter was not a key determinant of gamma pass rate performance, the combination of VSM model and delivery machine had a considerable effect on plan quality in SIMT treatments.

**Fig. 12:**
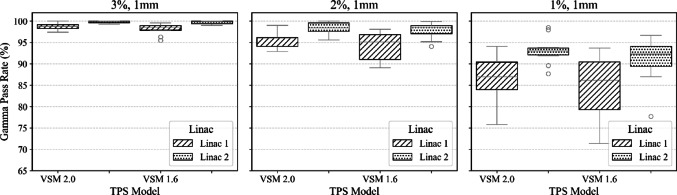
The boxplots illustrate the gamma pass rates for a cohort of stereotactic spine plans, comparing optimized and original beam models as measured with SRS MapCHECK. These boxplots present results for 3%, 1 mm; 2%, 1 mm; and 1%, 1 mm, calculated using VSM 2.0 and VSM 1.6 for LINAC 1 and LINAC 2

ANCOVA Analysis of data in Fig. 12 at the 1%, 1 mm gamma pass rate for spine cases was completed while using modulation complexity (MU/cGy) as a covariant. The effect of the TPS model on GPR changes depending on the LINAC delivered, (F(4, 34) = 59.10, *p* < 0.0001). In contrast, there were no statistically significant main effects for VSM model (*p* = 1.00), LINAC (*p* = 1.00), or modulation (*p* = 1.00). The interaction between model and modulation was also not significant (*p* = 0.26). These results suggest that while modulation did not significantly influence gamma pass rates in spine cases, machine-specific performance differences between beam models are a key driver of observed dosimetric outcomes. VSM 1.6 performed at least as well as VSM 2.0, particularly under stricter gamma criteria.

## Discussion

This study evaluated the performance improvements introduced in Monaco TPS version 6.2.2 with VSM 2.0, specifically the removal of MLC and jaw geometry dependent factors that required iterative tuning. This change is significant in how well the MLC is modelled for treatments that produce high dose gradients like complex stereotactic treatments. Our assessment comprised a direct comparison to VSM 1.6, interrogating MLC characterization via measurement with high resolution detector, OPF, ion chamber measurements in thorax phantom against LINAC 1, and how two LINACs perform considering a cohort of clinically relevant benchmarking intracranial and spine SRT cases.

### Output factor validation

Small-field dosimetry is often extrapolated within a TPS [3, 35], requiring validation and optimization for accurate dose calculations, particularly for stereotactic radiotherapy (SRT). In VSM 1.6, adjustments to leaf tip leakage and MLC offsets were historically required to improve small-field dosimetry accuracy. Without these refinements, dose discrepancies could compromise treatment accuracy, affecting clinical outcomes [36].

Our results demonstrate that VSM 2.0 achieves OPF values within 1.0% of measured data down to a 10 × 10 mm field size without the need for post modelling MLC adjustments, reinforcing the reliability of the Elekta AGL GPU model. Additionally, the OPF remained within expected tolerances for fields up to 400 × 400 mm, with no significant differences between the VSM 2.0 and 1.6 and LINAC 1.

### Point dose agreement in heterogenous phantom

Ionization chambers remain a source of truth as a measurement device due to their stability, precision and traceability. Completing 1583-point dose comparisons not only demonstrated equivalent or improved agreement with measurement using VSM 2.0, but it was also completed using a heterogeneous phantom to validate calculation in the presence of tissue like materials. In the analysis of the 1583 tests, the comparison between VSM 1.6 and VSM 2.0 demonstrated equivalent accuracy when compared with measurements from LINAC 1 (Table 5, 6, 7, 8, 9, 10). The average deviations from measured doses were smaller for the VSM 2.0, typically within a 1% margin. It is important to note that both VSM 1.6 and VSM 2.0 maintained dose differences within acceptable tolerances established by TD-1583. These results confirm that both models offer clinically acceptable point dose accuracy, with observed VSM differences falling within the expected range of statistical uncertainty inherent to Monte Carlo–based dose calculations.

**Table 8:** Case 6 measured dose values at selected reference points compared to calculated dose differences using two versions of VSM 1.6 (Monaco 6.1.4) and VSM 2.0 (Monaco 6.2.2)

	Ref point	Measured dose	VSM 1.6 (6.1.4)	VSM 2.0 (6.2.2)	Tol. %
		Dose (cGy)	%Diff	%Diff	
Case 6	3	236.1	− 0.3%	− 0.6%	3

**Table 9:** Case 7 measured dose values at selected reference points compared to calculated dose differences using two versions of VSM 1.6 (Monaco 6.1.4) and VSM 2.0 (Monaco 6.2.2)

	Ref point	Measured dose	VSM 1.6 (6.1.4)	VSM 2.0 (6.2.2)	Tol. %
		Dose (cGy)	%Diff	%Diff	
Case 7	3	37.5	2.0%	2.0%	4
	3	38.8	− 0.2%	− 0.5%	3
	3	38.9	− 1.3%	− 1.3%	3
	3	46.3	− 0.3%	− 0.6%	3
	3	43.5	0.0%	− 1.6%	3
	3	41.0	− 1.3%	− 0.6%	3
	3	38.2	1.1%	− 0.2%	4

**Table 10:** Case 8 measured dose values at selected reference points compared to calculated dose differences using two versions of VSM 1.6 (Monaco 6.1.4) and VSM 2.0 (Monaco 6.2.2)

	Ref point	Measured dose	VSM 1.6 (6.1.4)	VSM 2.0 (6.2.2)	Tol. %
		Dose (cGy)	%Diff	%Diff	
Case 8	3	94.8	− 0.6%	− 1.2%	3
	3	94.5	− 0.7%	− 0.7%	3
	3	99.2	− 0.1%	− 1.0%	3

### Validating the MLC characteristics

High-resolution diode array measurement provided a real time validation of MLC characterization of interleaf leakage and abutting field dosimetry. The FOURL (Fig. 6) and 7SegA (Fig. 7) fields were useful in validating the VSM 2.0, detailing the improved agreement between measured and calculated dose distributions, as demonstrated by higher gamma pass rates (Table 4). The use of real-time high-resolution detectors provides a consistent tool to interrogate delivery systems when compared to film, which can pose a challenge due to handling and processing. One of the marked improvements observed is the match of tongue and groove characteristics between MLC, even at tighter criteria. Historically, VSM 1.6 required aggressive tuning of the “Leaf Groove Width” parameter to match film or array measurements, this approach has led to overfitting and still failed to fully correct discrepancies between measured and calculated [10].

### Validating clinical cases

Three clinical plan types: single-target(s), a SIMT brain and spine plans were delivered to two clinically beam-matched Elekta LINACs. In all cases the Accelerated Go live VSM 2.0 model maintained or improved GPR performance, particularly under high-modulation and small-segment planning conditions, without requiring manual geometric tuning of the MLC or jaw configuration. Where other studies required adjustment to see improvements to GPR for validation cases with VSM 1.6 [7, 8]. To quantitatively assess model performance, gamma analysis was performed using an absolute dose comparison with a global γ calculation and a 10% threshold for low dose cut-off. Clinically, within our institution, a reference criterion of 3%/1 mm is routinely applied, measured as a true composite [37].

In the single-target dataset, VSM 2.0 produced slightly higher gamma pass rates than VSM 1.6, particularly for plans with smaller average segment sizes. An ANCOVA indicated that segment size (cm^2^) was not a statistically significant covariate, but a modest trend toward lower pass rates in VSM 1.6 for smaller segments was observed. This suggests that while VSM 2.0 may not be explicitly segment-size dependent, more accurate modelling of MLC transmission and leakage may lead to the improvements seen in small, highly modulated plans. The multimet analysis showed a more variable pattern in gamma pass rate performance, particularly when targets were located at greater distances from isocentre. Our study had targets up to 5.0 cm away from isocentre, similar to Saenz et al. [38], VSM 2.0 demonstrated improvement in maintaining high gamma agreement across the range of off-axis targets. ANCOVA revealed a significant interaction between VSM model and distance from isocentre, suggesting that the enhanced modelling in VSM 2.0 more accurately accounts off axis deliveries dictated by target location. This is especially relevant in single-isocenter, multiple-target SRS, where geometric inaccuracies can compound, and warrants further investigation with a larger cohort of SIMT cases.

The spine cases showed the highest range in GPR at 1%, 1 mm, however, VSM 2.0 consistently outperformed VSM 1.6 in plans with high modulation indices (MU/cGy). Higher values for the MU factor are correlated with more complex deliveries [39] (Tables 2 and 3). The ANCOVA results showed a statistically significant interaction between VSM model and LINAC, highlighting the improved reproducibility of VSM 2.0 between matched machines. Interestingly, modulation was not a significant covariate in VSM 2.0 plans, whereas pass rate degradation was evident in high-modulation VSM 1.6 plans, indicating a reduced sensitivity to delivery complexity in the updated model. Our inference would be that the modelling of MLC in VSM 2.0 leads to better agreement between LINAC and TPS at the tighter criteria. The variability between LINAC 1 and 2 likely indicates that residual discrepancies may originate from mechanical or calibration differences between machines, rather than from the beam model. This observation aligns with results by Goodall et al. [21], who reported that GPR variability across a fleet of beam-matched Elekta LINACs delivering SBRT spine treatments was moderately correlated with plan complexity (MU/cGy) but often linked to specific LINACs rather than systematic errors in TPS modelling. They concluded that some LINACs consistently underperformed at stricter gamma criteria, despite use of a common beam model, implicating LINAC-specific delivery characteristics, such as MLC calibration, mechanical sag, or gantry rotation effects, as potential sources of inconsistency.

Tightening the gamma criteria serves as a method to detect systematic differences between the TPS-calculated and measured fluence profiles on the LINAC [24]. Both VSM models meet institutional standards for 2D planar assessment, demonstrating > 95% with 3%- and 1-mm criteria and 10% dose threshold. Our study has shown VSM 2.0 overall improves the calculation accuracy when very small segments are delivered during complex treatments. Figures 10, 11, 12 illustrate a systematic improvement in VSM 2.0 when compared to VSM 1.6. Although the measured case cohort is limited, it consists of clinically relevant deliveries, serving as a benchmark for performance across a matched cohort of LINACs. These cases are carefully curated, with planning templates that mitigate the generation of highly modulated plans, and function as a quality assurance tool within our institution.

The improvement ushered in by VSM 2.0 will allow for one robust beam model to be deployed across multiple LINACs and institutions, ensuring consistency for complex stereotactic treatments. We observed optimal performance for simple OPF and ionization chamber measurements in heterogenous phantom, reduced inter-LINAC variation for two matched LINACs, and an improved model fidelity in small-field, off-axis and high modulation deliveries. The elimination of manually tuned geometric parameters simplifies commissioning and reduces the risk of modelling errors, particularly in complex plans. Future research will focus on completing a study to understand the impact of introduced errors in plans within VSM 2.0 on deliveries across a cohort of matched LINACs for intracranial SRS cases. As LINAC modelling within the TPS continues to evolve, these efforts will help build confidence in distributing complex treatment cases across a well-matched LINAC cohort.

## Conclusions

This study demonstrates that VSM 2.0 in Monaco TPS (version 6.2.2) improves the accuracy and consistency of dose calculations for complex stereotactic treatments compared to its predecessor, VSM 1.6. By removing the need for geometry-dependent factors, VSM 2.0 offers enhanced modelling of the multileaf collimator and jaws, reducing the reliance on iterative tuning to achieve a high-quality beam model.

Across the tested indications (single-target brain SRS, multi-target SRS, and spine SABR) VSM 2.0 provided robust gamma pass rates and reduced LINAC-to-LINAC variability. From the cohort of plans, covariate influences such as segment size, modulation complexity and target location showed limited influence, but detailed a reduced dependency when compared to VSM2.0. These initial results support the clinical implementation of a single, shared beam model across beam-matched LINACs, enabling more standardized and efficient workflows for stereotactic treatment delivery for the AGL 6FFF model.

VSM 2.0 represents a meaningful advancement in beam modelling within Monte Carlo-based TPS environments, offering improved dosimetric performance, operational simplicity, and the potential to support automation and scaling of high-precision radiotherapy programs across institutions.

## Data Availability

The datasets generated and or analyzed during the current study are available from the corresponding author on reasonable request.

## References

[1] Ma CMC et al (2020) Beam modeling and beam model commissioning for Monte Carlo dose calculation-based radiation therapy treatment planning: report of AAPM Task Group 157. Med Phys 47(1):e1–e18 31679157 10.1002/mp.13898

[2] Das IJ et al (2008) Accelerator beam data commissioning equipment and procedures: report of the TG-106 of the therapy physics committee of the AAPM. Med Phys 35(9):4186–4215 18841871 10.1118/1.2969070

[3] Smilowitz JB et al (2015) AAPM medical physics practice guideline 5.a.: commissioning and QA of treatment planning dose calculations—megavoltage photon and electron beams. J Appl Clin Med Phy 16(5):14–34 10.1120/jacmp.v16i5.5768 PMC5690154 26699330

[4] Gholampourkashi S et al (2019) Monte Carlo and analytic modeling of an Elekta Infinity LINAC with agility MLC: investigating the significance of accurate model parameters for small radiation fields. J Appl Clin Med Phys 20(1):55–67 30408308 10.1002/acm2.12485 PMC6333188

[5] Hillman Y et al (2018) Refinement of MLC modeling improves commercial QA dosimetry system for SRS and SBRT patient-specific QA. Med Phys 45(4):1351–1359 29431865 10.1002/mp.12808

[6] Roche M et al (2018) Agility MLC transmission optimization in the Monaco treatment planning system. J Appl Clin Med Phys 19(5):473–482 29959822 10.1002/acm2.12399 PMC6123174

[7] Snyder M et al (2016) Modeling the agility MLC in the Monaco treatment planning system. J Appl Clin Med Phys 17(3):190–202 27167277 10.1120/jacmp.v17i3.6044 PMC5690908

[8] Inc., IMS (2013) Monaco post modeling adjustment of MLC parameters. (Document ID: LRMMON0003)

[9] Thewes L, Eckl M, Schneider F (2023) Transmission probability filter optimization for agility MLC in Monaco treatment planning system. J Appl Clin Med Phys 24(9):e14105 37494135 10.1002/acm2.14105 PMC10476981

[10] Hernandez V et al (2022) Challenges in modeling the Agility multileaf collimator in treatment planning systems and current needs for improvement. Med Phys 49(12):7404–7416 36217283 10.1002/mp.16016 PMC10092639

[11] Saez J et al (2023) Universal evaluation of MLC models in treatment planning systems based on a common set of dynamic tests. Radiother Oncol 186:109775 37385376 10.1016/j.radonc.2023.109775

[12] Van Esch A et al (2022) Testing of an enhanced leaf model for improved dose calculation in a commercial treatment planning system. Med Phys 49(12):7754–7765 36190516 10.1002/mp.16019

[13] Panagi R, Caines R, Rowbottom CG (2025) Dosimetric sensitivity of an enhanced leaf model (ELM) for individual versus averaged machines. J Appl Clin Med Phys 26:e14621 39902660 10.1002/acm2.14621 PMC11969076

[14] Rijken J et al (2019) Distributive quality assurance and delivery of stereotactic ablative radiotherapy treatments amongst beam matched linear accelerators: a feasibility study. J Appl Clin Med Phys 20(4):99–105 30883010 10.1002/acm2.12567 PMC6448346

[15] Muñoz L et al (2021) Consistency of small-field dosimetry, on and off Axis, in Beam-matched LINACs used for stereotactic radiosurgery. J Appl Clin Med Phys 22:185–193 33440049 10.1002/acm2.13160 PMC7882112

[16] Muñoz L et al (2020) Assessing small-field output factors using a 2D monolithic diode array on a beam-matched Elekta linear accelerator. J Phys Conf Ser 1662:012024

[17] Klein EE et al (2009) Task group 142 report: quality assurance of medical acceleratorsa. Med Phys 36(9):4197–4212 19810494 10.1118/1.3190392

[18] Krauss RF et al (2023) AAPM medical physics practice guideline 8.b: linear accelerator performance tests. J Appl Clin Med Phys 24(11):e14160 37793084 10.1002/acm2.14160 PMC10647991

[19] Norvill C et al (2021) Investigation of Elekta AQUA software for kilovoltage to megavoltage radiation isocenter coincidence. Phys Eng Sci Med 44(3):667–675 34033014 10.1007/s13246-021-01014-1

[20] Cirino E et al (2025) AAPM-RSS medical physics practice guideline 9.b: SRS-SBRT. J Appl Clin Med Phys 26(4):e14624 40071780 10.1002/acm2.14624 PMC11969102

[21] Goodall SK et al (2022) Matched LINAC stereotactic radiotherapy: an assessment of delivery similarity and distributive patient-specific quality assurance feasibility. J Appl Clin Med Phys 23:e13652 35570398 10.1002/acm2.13652 PMC9680571

[22] Goodall SK, Ebert MA (2020) Recommended dose voxel size and statistical uncertainty parameters for precision of Monte Carlo dose calculation in stereotactic radiotherapy. J Appl Clin Med Phys 21:120–130 33124741 10.1002/acm2.13077 PMC7769395

[23] Stedem AK et al (2024) Systematic evaluation of spatial resolution and gamma criteria for quality assurance with detector arrays in stereotactic radiosurgery. J Appl Clin Med Phys 25(2):e14274 38265979 10.1002/acm2.14274 PMC10860444

[24] Geurts MW et al (2022) AAPM medical physics practice guideline 5.b: commissioning and QA of treatment planning dose calculations—Megavoltage photon and electron beams. J Appl Clin Med Phys 23(9):e13641 35950259 10.1002/acm2.13641 PMC9512346

[25] TecDoc I (2008) 1583: commissioning of radiotherapy treatment planning systems: testing for typical external beam treatment techniques. International Atomic Energy Agency, Vienna

[26] Ezzell GA et al (2009) IMRT commissioning: multiple institution planning and dosimetry comparisons, a report from AAPM Task Group 119. Med Phys 36(11):5359–5373 19994544 10.1118/1.3238104

[27] TRS, I. (2017) 483‐dosimetry of small static fields used in external beam radiotherapy. IAEA, Vienna 10.1002/mp.13208 30247757

[28] Goodall SK, Rowshanfarzad P, Ebert MA (2023) Correction factors for commissioning and patient specific quality assurance of stereotactic fields in a Monte Carlo based treatment planning system: TPS correction factors. Phys Eng Sci Med 46(2):735–745 37022612 10.1007/s13246-023-01246-3 PMC10209265

[29] Paddick I et al (2023) Benchmarking tests of contemporary SRS platforms: have Technological developments resulted in improved treatment plan quality? Pract Radiat Oncol 13:451–459 10.1016/j.prro.2023.05.005 37290672

[30] Park HS et al (2016) Changing practice patterns of Gamma Knife versus linear accelerator-based stereotactic radiosurgery for brain metastases in the US. J Neurosurg 124(4):1018–1024 26473783 10.3171/2015.4.JNS1573

[31] Timmerman RD, Herman J, Cho LC (2014) Emergence of stereotactic body radiation therapy and its impact on current and future clinical practice. J Clin Oncol 32(26):2847 25113761 10.1200/JCO.2014.55.4675 PMC4152712

[32] Halvorsen PH et al (2017) AAPM-RSS medical physics practice guideline 9. a. for SRS-SBRT. J Appl Clin Med Phys 18(5):10–21 28786239 10.1002/acm2.12146 PMC5874865

[33] Mynampati DK et al (2012) Application of AAPM TG 119 to volumetric arc therapy (VMAT). J Appl Clin Med Phys 13(5):108–116 10.1120/jacmp.v13i5.3382 PMC5718241 22955639

[34] Rose MS et al (2020) Multi-institution validation of a new high spatial resolution diode array for SRS and SBRT plan pretreatment quality assurance. Med Phys 47:3153–3164 32215929 10.1002/mp.14153

[35] Lechner W et al (2018) A multinational audit of small field output factors calculated by treatment planning systems used in radiotherapy. Phys Imaging Radiat Oncol 5:58–63 33458370 10.1016/j.phro.2018.02.005 PMC7807586

[36] Kairn T et al (2016) Effects of inaccurate small field dose measurements on calculated treatment doses. Australas Phys Eng Sci Med 39(3):747–753 27380010 10.1007/s13246-016-0461-y

[37] Miften M et al (2018) Tolerance limits and methodologies for IMRT measurement-based verification QA: recommendations of AAPM Task Group No. 218. Med Phys 45(4):e53–e83 29443390 10.1002/mp.12810

[38] Saenz D et al (2020) Robustness of single-isocenter multiple-metastasis stereotactic radiosurgery end-to-end testing across institutions. J Radiosurg SBRT 7(3):223 PMC8055241 33898086

[39] Nguyen M, Chan GH (2020) Quantified VMAT plan complexity in relation to measurement-based quality assurance results. J Appl Clin Med Phys 21(11):132–140 33112467 10.1002/acm2.13048 PMC7700925

